# Autistic Employees’ Technology-Based Workplace Accommodation Preferences Survey—Preliminary Findings

**DOI:** 10.3390/ijerph20105773

**Published:** 2023-05-09

**Authors:** Michał T. Tomczak, Paweł Ziemiański

**Affiliations:** Faculty of Management and Economics, Gdańsk University of Technology, 80-233 Gdańsk, Poland; pawel.ziemianski@pg.edu.pl

**Keywords:** autism, neurodiversity, technology-based accommodations, assistive technology, work environment, quantitative research, survey

## Abstract

Background: There has been an increase in the number of research studies focused on the design of accommodations aimed at improving the well-being and work performance of autistic employees. These accommodations took various forms; some of them were based on modification of management practices, for example, support in the area of effective communication, or involved modifications to the physical working environment aimed at limiting sensory vulnerabilities. Many of these solutions were based on digital technology. Methods: This quantitative research aimed to learn about the opinions of the autistic respondents as potential end users and their assessment of the proposed solutions within four main challenge areas: (1) effective communication; (2) time management, task prioritizing, and organization of work; (3) stress management and emotion control; and (4) sensory sensitivities. Results: Respondents gave the highest ratings to solutions aimed at limiting overstimulation and a flexible approach toward working time, support of a job coach, remote work, and support by allowing electronic-mediated communication based on non-direct contact. Conclusions: The results can be the starting point for further research on the highest rated solutions dedicated to improving working conditions and the well-being of autistic employees and can be an inspiration for employers who plan to introduce such solutions.

## 1. Introduction

In recent years, the issue of labour market integration [1] and managing diverse teams that include neurodivergent employees [2] has gained importance. Some have even suggested workplace success strategies for employees with autism as a new frontier for Human Resource development [3].

Thus, there has been an increase in the amount of research focused on the design of intervention programs [4,5,6,7,8,9]. and various workplace accommodations [10,11,12,13]. All of them were aimed at improving the work performance and the well-being of autistic employees in the workplace by providing support to overcome challenges such as interpersonal communication, social reciprocity, and sensory sensitivity, which are specific to many individuals with this condition [14].

Specific actions, on the one hand, should be tailored to the individual needs of the neurodivergent employee, for example, taking into account his or her preferred communication style or the need to reduce stimuli from the environment. On the other hand, they should not impede the work of other employees but at the same time be available to all employees if they are interested. According to Petty et al. [15], reasonable adjustments are those that are having a positive impact on autistic employees, both on their well-being and work output, without being detrimental to neurotypical employees or the organization.

### 1.1. Prior Studies on Technology-Based Workplace Accommodations

Technology-based workplace accommodations are modifications to the physical work environment or human resource management practices using technology. Importantly, many of these solutions were based on digital technology [16,17,18,19], such as computer applications or mobile devices. Some of them have involved technology for the general population, for example, by optimizing the available solutions addressed to conduct work in remote form [20,21,22]. Others were strictly within the realm of assistive technology (AT), tailored to employees with autism as a specific group among the neurodivergent community, to meet their particular needs and characteristics and support them in overcoming the above-mentioned challenges [23].

These accommodation proposals took various forms: some of them were based on developing tailored recruitment practices, improvements in communication, and social skills development [6,7,24,25]; remote/hybrid work [21,22,26,27]; stress level and notification of the increase in stress level [28,29]; limitation of sensory overload [30,31,32,33,34]; improvement of well-being [10,35,36,37,38,39,40,41]; or prevention of burnout [42,43].

Significantly, only a handful of studies involved research on larger samples of autistic community representatives, and most referred to qualitative methods exploring expert and autistic self-advocate opinions or case studies. Our study differs from others in that, first of all, a quantitative methodology was used, which has been rare in previous studies on the subject. Second, we were able to reach a fairly large sample of people who are part of the autistic community.

### 1.2. The Aim of Research

This research aims to explore the opinions of autistic respondents as potential end users, about their evaluation of the selected technology-based solutions identified as a result of the previous own research [41], and identify the most beneficial accommodation in the opinion of members of the autistic community.

This is especially important in the case of Poland, where autism incidence rates are persistently lower compared to western Europe and North America [44] and a significant proportion of autistic individuals likely remain undiagnosed or do not opt to disclose their condition. This translates into low levels of awareness among employers and colleagues, and a small number of inclusion initiatives and recruitment programs tailored for neurodivergent communities. In this situation, any support is greatly appreciated.

## 2. Methods

### 2.1. Procedure

As a result of the analysis of the current literature and prior research conducted by the first author, the main challenges within the work environment were identified, including the following:

(1) Effective communication [25,34,45,46,47];
(2) Time management, task prioritization, and organization of work [41,48,49];
(3) Stress management and emotional control [28,29].
(4) Sensory sensitivities [34,41].

Second, in response to the challenges listed above, a set of 12 technology-based workplace accommodations was identified that fit the 4 challenge areas [41] and then presented for evaluation to a group of autistic individuals to determine their opinions on the proposed solutions.

The respondents were asked to rate selected proposals for technology-based workplace accommodations on a 5-point scale (1—very bad, 2—bad, 3—neither good nor bad, 4—good, 5—very good). These proposals are presented below and were all rated on the same scale, 1 to 5.

1. Electronic forms of communication (instant messaging, chat rooms, discussion forums, chatbots);
2. Speech synthesizers (text-to-speech);
3. Flexible working time;
4. Remote work (cloud computing, virtual teams, home office);
5. Computer and mobile applications facilitating work-time control and task prioritization (clear instructions and work structure);
6. Ongoing support by a job coach/consultant;
7. Organization of work using solutions employing virtual reality (avatars, virtual office);
8. Organization of work using gamification;
9. Stress measurement combined with dynamic customization of office environment parameters (temperature, humidity, noise, smell, sunlight exposure);
10. Stress level measurement with up-to-date stress level increase notifications (wrist-worn device);
11. Arrangement of office space according to the needs of autistic employees (chill rooms, avoiding bright colours, working with headphones);
12. Personalization of the workplace according to the individual preferences of users (furniture and office equipment; adjusting the parameters of the environment: temperature, humidity, noise, smell, sunlight exposure).

This was followed by additional questions on gender, professional status, job experience, level of education, employment sector, and self-assessment of competencies in the use of digital technologies.

Due to the exploratory nature of the study, no preliminary hypotheses were formulated. For examining the views of respondents, it was decided to use the research method based on non-direct contact, computer-assisted web interview (CAWI). The online questionnaire was carried out using Qualtrics XM (Qualtrics International Inc., Seattle, WA, USA) and the data were analysed using IBM SPSS Statistics 25 (IBM Corp., Armonk, NY, USA).

The study was carried out according to the ethical guidelines and procedures of the Gdańsk University of Technology (No. 303/2011).

### 2.2. Participants

The research sample was based on non-random, purposive selection and included adults diagnosed with autism (113 women and 27 men). The link to the survey was distributed through closed forums on social media and websites dedicated to autistic adults, with permission from the forum/website administrator. Additionally, with the use of snowball sampling, when the invitation to participate in the study was individually sent by email to the representatives of the autistic community in Poland, they were then involved in the implementation of the study by disseminating the survey questionnaire. The method performed for participant recruitment did not allow us to obtain a representative sample. However, the sample size was quite large for a study involving representatives of the unexplored population of autistic employees in Poland.

Women were significantly predominant in the research sample, the vast majority of respondents were working at the time of participation in the study, and a third of them worked in the IT sector. Most of the respondents had professional experience of more than five years and a higher education (bachelor’s or master’s degree). In particular, the vast majority (89.3%) of the respondents described their self-assessment of their competencies in the use of digital technologies as ‘good’ or ‘very good’. Detailed information on the research sample is shown in Table 1. Other specific data on socioeconomic status were not recorded.

### 2.3. Statistical Analysis

Descriptive statistics were computed to characterize the research sample (percentages) and to verify the assessment of proposals for technology-based workplace accommodations in the entire sample of participants and within subgroups (means and standard deviations). Further statistical analysis involved comparing how the proposals were assessed within the subgroups of participants—independent-sample Student’s *t*-test was used. Additionally, Spearman’s rank-order correlation coefficient was computed to verify the relationship between the proposals’ assessment and the self-assessment of digital competencies in the entire sample. A non-parametric Spearman’s correlation coefficient was selected due to the distribution of the self-assessment of competencies in the use of digital technologies in the research sample (almost 90% of answers fell within the “good” or “very good” category). The results of the analyses and their discussion are presented in the following sections of the article.

## 3. Results

In solutions that support effective communication, autistic respondents gave the highest rating (4.25) to electronic forms of communication, for example, email, online communicators, or chats.

Regarding time management, setting priorities, and organization of work, four solutions received the highest scores: flexible working time (4.66); remote work (4.43); computer and mobile applications facilitating work-time control and task prioritization (4.31); and ongoing support from a job coach (4.24).

In terms of stress management, the highest rating (3.84) was given to stress level measurement combined with dynamic customization of the office environment psychical parameters. Still, it was lower than in the case of the assessments of other challenge areas.

Finally, as part of the solutions to overcome the effects of sensory sensitivity, respondents highly rated both office space arrangements based on the needs of autistic individuals (4.79) and personalization of the workplace according to the individual preferences of the users (4.79).

The detailed responses and the full list of the proposed solutions evaluated by the respondents are presented in Table 2.

We compared the assessment of the selected proposals for improvement within two research participants’ subgroups: less experienced persons with experience of up to 5 years and more experienced persons with over 5 years of experience (in this comparison, the answers of people who have never worked were not included). It was discovered that the only aspect where the difference was statistically significant pertained to the use of computer and mobile applications that facilitate work-time control and task prioritization. The result obtained indicates that, in the case of less experienced people, this improvement may be of even greater importance.

A further comparison between the subgroups of participants based on their education level was also performed. Participants with a higher education were included in one group and those with primary, vocational, and secondary levels were included in the second group. Only one proposed improvement was assessed differently by the members of those groups. Again, these were applications facilitating work-time control and task prioritization, which were assessed as more important by the group of participants with lower education levels (M = 4.54, SD = 0.65) than the group of people with a higher education (M = 4.20, SD = 0.92), *t* = 2.308, *p* < 0.05). All other improvements were assessed similarly.

Naturally, there are multiple ways in which research participants could be divided into subgroups. In the present research, we compared the groups of people differentiated by the length of work experience and education level. The rationale behind the first categorization was related to the fact that for people with autism, the early period of professional activity may be particularly important and decisive in developing motivation and self-confidence in their career. It is thus crucial to explore whether they view particular proposals for improvements differently. Exploring this topic could also provide practical conclusions for employers. Interestingly, only one significant difference was found. This result allows us to draw a cautious conclusion that computer and mobile applications that facilitate work-time control and task prioritization are more crucial for less experienced people who are, to a greater extent, in the process of learning how to conduct work and perform their tasks. The rest of the proposed improvements seem to be universally important to autistic employees.

Similarly, the assessment of applications facilitating work-time control and task prioritization was different in the subgroups created with regard to education. People with a higher education level considered them less important than those with lower education levels, which may be related to the fact that the former group is more likely to become knowledge workers for whom having individual control over one’s priorities and tasks is important. The offered explanation is plausible but undoubtedly requires further investigation.

The results of the statistical analyses should be considered exploratory and further analyses should be conducted. For example, the length of work experience may also be related to the position within the organization (i.e., being a manager), and thus the result obtained in the current study requires further verification. Additionally, variables such as the sector of employment and belonging to a particular generation that is active in the labour market should be further investigated as possible moderating variables.

We also verified how the self-assessment of competency in the use of digital technologies was related to the assessment of selected proposals for improvement (Spearman correlation coefficient was computed). Six correlations indicated in the last column of Table 2 were statistically significant. They are all positive, and even though correlation does not imply causation, it can be cautiously proposed that those six improvements may be more important for those autistic employees who are more digitally competent.

## 4. Discussion

Despite the motivation and proven abilities of many autistic people, their employment rate remains low [40] throughout the world [5,50]. For example, in the United States, the unemployment/underemployment rate for individuals on the autism spectrum is greater than 90% [51], and in the United Kingdom only 20% of autistic people are employed [52]; however, there are no such data for Poland. A good opportunity to reverse this unfavourable phenomenon may be a digital transformation of organizations [53,54]. Importantly, many autistic individuals work in the IT sector or assume job roles related to handling digital technologies [55]. Digital technologies strongly complement neurodiversity initiatives, and various technologies can be leveraged to improve the inclusiveness of recruitment, training, digital supervision, flexible workspaces, and mental health policies [18,34,56].

Although we recognize the various benefits of using digital technology in the inclusion of neurodiverse people, including those with autism, we should also be aware of some risks. The widespread use of information and communication technologies in modern society can be related to the phenomenon of techno-stress [57], and high-risk situations should be recognized as soon as possible. Additionally, there is a risk of stigmatization of assistive technology users [58,59]. Finally, there may be challenges in accepting the technological solutions proposed by users [60]. Therefore, in order to avoid the occurrence of the above risks, the implementation of technology in the process of inclusion of neurodivergent people should first be preceded by an in-depth analysis of the needs of individual employees on each job, in terms of the need for specific support solutions, such as those that improve communication, work organization, or reduce stimuli. Second, all employees should be trained both in the functionality of technological solutions and prudent use of them, while maintaining the principle of work–life balance.

The solutions rated highest by the respondents can positively affect the employment and well-being of autistic individuals. Reducing nuisance stimuli from the environment by rearranging the workplace according to individual preferences, e.g., allowing work with headphones or enabling dynamically customizing ambient environmental parameters (e.g., temperature, humidity, noise, smell, sunlight exposure) can provide important support in the context of sensory vulnerabilities. Next, providing a flexible working time, including remote working and support in the area of time control and prioritization. There are already options available on the market, such as ‘Brain in Hand’ [61] to support time management and prioritization, or ‘Life Sherpa’ [62] to monitor work activities with online access to mentor/coach/work coach support. Finally, it will also be important to support more effective communication between neurotypical and neurodivergent employees. An example would be IBM Watson Content Clarifier [63], an application aimed at supporting reading comprehension. The above solutions can have a positive impact not only on work performance, but also on the improvement of well-being, and countering job burnout [43]. Next, the effectiveness of adjustments should be objectively evaluated and promoted to determine what works for whom and how the quality of the provisions can be benchmarked [64].

Our result may be considered through the lens of a social model of disability [65], according to which the barriers faced by people with disabilities are not just the result of impairments, but also a social construct because structures and environments are created by others [66]. As a result, by offering support, e.g., in the form of solutions to overcome challenges, we are simultaneously contributing to the elimination of barriers that stand in the way of successful employment for people with autism.

### 4.1. Implications

Researchers and practitioners must work together to increase the impact of occupational safety and health innovations [67]. We believe that the results obtained can not only be the starting point for further research on the highest rated solutions and accommodations dedicated to improving the working conditions and the well-being of autistic employees, but can also be an inspiration for employers planning to introduce such solutions. Identifying supportive solutions that are rated highest in terms of usefulness by people with autism is critical, as it allows efforts to be focused and resources to be committed to developing solutions that can have the greatest positive impact.

### 4.2. Limitations

The main limitation of the presented research, which is only a preliminary study, is the limited representativeness due to the non-random sample selection and as a consequence of the asymmetric structure of the research sample in terms of gender and only one country of origin of the respondents. Despite this, we believe the results obtained are a valuable source of knowledge, as the challenges faced by people with autism around the world are similar. The proposals for supportive solutions are universal and can be addressed to employees on the spectrum from different countries and cultural backgrounds. The third limitation is the use of self-reported data, as is the case with surveys. Our survey involved identifying the opinions of the respondents in the area of evaluating selected support solutions, so we rely on the declarations of the representatives of the autistic community. On this basis, further research can be designed, for example, in the form of experiments in which the highest-rated solutions would be tested.

## 5. Conclusions

It appears that among the proposed solutions, autistic respondents gave the highest ratings to those aimed at limiting the overstimulation by external stimuli, and then pointing to solutions in the area of work organization, particularly a flexible approach toward working time, support of a job coach, remote work, and support by allowing an electronic form of communication based on non-direct contact.

The use of computer and mobile applications that support time management, task prioritization, and work organization is more important for less experienced employees (working up to 5 years) and those with lower education levels. Some improvements may be more important for those autistic employees who have self-assessed themselves as digitally competent.

More research in these directions should be carried out to implement the solutions mentioned above in practice, test them in real working conditions, and then evaluate their actual effectiveness.

## Figures and Tables

**Table 1:** Detailed information on the research sample, *N* = 140.

Characteristics		% of Respondents
Gender	Female	80.7
	Male	19.3
Professional status	Employed	85.7
	Unemployed	14.3
Seniority (among employed)	Less than 1 year	9.1
	1 to 5 years	26.7
	More than 5 years	64.2
Level of education	Higher	65.7
	Secondary	30
	Vocational	0.7
	Primary	3.6
Self-assessment of competencies in the use of digital technologies	Very bad	0
	Bad	0.7
	Average	10
	Good	42.1
	Very good	47.2
Employment sector	IT	31.6
	Other	68.4

**Table 2:** Results of statistical analyses, *N* =140.

Proposal for Improvement in a Given Area of Challenge Assessed by Respondents on a 5-Point Scale: 1—Very Bad, 2—Bad, 3—Neither Good Nor Bad, 4—Good, 5—Very Good	Means (All Participants), *N* = 140	Standard Deviation	Means—Less Experienced Group; (Up to 5 Years), *N* = 43	Means—More Experienced Group; (Over 5 Years), *N* = 77	Spearman Correlation (rho) with Self-Assessment of Digital Competency
**Effective Communication**					
Electronic forms of communication	4.25	0.85	4.40	4.22	0.28 **
Speech synthesizers	2.97	1.00	2.74	3.03	−0.10
**Time Management, Task Prioritizing and Work Organizing**					
Flexible working time	4.66	0.72	4.79	4.65	0.17 *
Remote work	4.43	0.86	4.49	4.48	0.31 **
Computer and mobile applications facilitating work-time control and task prioritization	4.31	0.85	4.63 +	4.09 +	0.08
Ongoing support by a job coach/consultant	4.24	0.77	4.30	4.21	−0.15
Organization of work using solutions employing virtual reality	3.13	0.94	3.02	3.25	0.19 *
Organization of work using gamification	3.09	1.00	3.09	3.10	−0.01
**Stress Management and Emotion Control**					
Stress measurement combined with dynamic customization of office environment parameters	3.84	1.16	3.81	3.77	0.09
Stress level measurement with up-to-date stress level increase notifications	3.10	1.31	3.12	3.03	0.03
**Sensory Sensitiveness**					
Arrangement of office space according to the needs of autistic employees	4.79	0.47	4.79	4.79	0.17 *
Personalization of the workplace according to the individual preferences of users	4.79	0.51	4.86	4.78	0.18 *

* Note: + difference between means in subgroups significant at *p* < 0.001, *t*-Test value (118) = 4.843. * *p* < 0.05, ** *p* < 0.01.

## Data Availability

The data set used and analysed during the current study is available from the corresponding author upon reasonable request.
